# Bioremediation of cooking oil waste using lipases from wastes

**DOI:** 10.1371/journal.pone.0186246

**Published:** 2017-10-26

**Authors:** Clarissa Hamaio Okino-Delgado, Débora Zanoni do Prado, Roselaine Facanali, Márcia Mayo Ortiz Marques, Augusto Santana Nascimento, Célio Junior da Costa Fernandes, William Fernando Zambuzzi, Luciana Francisco Fleuri

**Affiliations:** 1 Chemistry and Biochemistry Department, Institute of Biosciences, São Paulo State University (UNESP), Botucatu, SP, Brazil; 2 Agronomic Institute (IAC), CEP, Campinas, SP, Brazil; MJP Rohilkhand University, INDIA

## Abstract

Cooking oil waste leads to well-known environmental impacts and its bioremediation by lipase-based enzymatic activity can minimize the high cytotoxic potential. In addition, they are among the biocatalysts most commercialized worldwide due to the versatility of reactions and substrates. However, although lipases are able to process cooking oil wastes, the products generated from this process do not necessarily become less toxic. Thus, the aim of the current study is to analyze the bioremediation of lipase-catalyzed cooking oil wastes, as well as their effect on the cytotoxicity of both the oil and its waste before and after enzymatic treatment. Thus, assessed the post-frying modification in soybean oil and in its waste, which was caused by hydrolysis reaction catalyzed by commercial and home-made lipases. The presence of lipases in the extracts obtained from orange wastes was identified by zymography. The profile of the fatty acid esters formed after these reactions was detected and quantified through gas chromatography and fatty acids profile compared through multivariate statistical analyses. Finally, the soybean oil and its waste, with and without enzymatic treatment, were assessed for toxicity in cytotoxicity assays conducted *in vitro* using fibroblast cell culture. The soybean oil wastes treated with core and frit lipases through transesterification reaction were less toxic than the untreated oils, thus confirming that cooking oil wastes can be bioremediated using orange lipases.

## Introduction

Lipids and carbohydrates are the main waste constituents generated by the food industry. In addition, they cause damages to the environment due to the formation of oily films on aquatic surfaces, which disrupts oxygen diffusion, and cloggings mainly caused by the emulsification with organic matter, and for oil methanization, which worsens the greenhouse effect [1–2]. Since, one liter of lipid waste can compromise approximately 1 million liters of natural water, mainly when the oil is subjected to elevated temperatures, since even oils considered healthy at room temperature can become toxic when they are heated [3–4]. Thus, investigating post-heating oil modification, as well as the possibilities to reduce their toxicity, is essential to the food industry, since these issues refer to a typical oil-processing situation.

Frying-based food is among the processes able to modify the structure of oils, as well as to increase their toxicity. The compounds resulting from the frying process have been associated with diseases such as cancer, Alzheimer and Parkinson [5], but their effects on biological models are poorly understood. Thus, we believe that by understanding the changes in the structure of oils subjected to heating, as well as their behavior in biological models, we will be able to suggest processes able to minimize the negative effect resulting from the consumption of these products, as well as the environmental impact caused by the generated wastes.

In this context, important studies somehow have reported the applicability of lipase activities (EC 3.1.1.3 triacylglycerol acylhydrolases) in lipid waste bioremediation. Since, these enzymes catalyzed a wide variety of lipid transformations, due to their broad substrate affinity, high stability towards temperatures and solvents [6]. Importantly, they have emphasized technical issues, which should be taken into account such as specificity (allows converting the wastes into non-toxic by-products), higher yield, possibility of applying the method to wastes showing high or low pollutant contents; operation under mild conditions and, consequently, energy-cost reduction [2, 7–9]. The aforementioned studies have shown that the composition of fatty acids ester (FAE) was modified by lipase-catalyzed reactions. However, these studies did not indicate whether this modification affected the toxicity of these wastes. Thus, assessing the cytotoxicity of those oils before and after bioremediation would help supporting the investigation about the role played by this process in the toxicity of lipid wastes.

Living systems are exposed to numerous harmful substances able to cause cell damage. Thus, the ability to neutralize such molecules is essential to provide adequate metabolic functioning; metabolism itself has developed mechanisms to identify and degrade or turn these molecules into inert compounds. Thus, bioremediation emerges as biotechnological application of these processes aiming at neutralizing target molecules; it can be accomplished through the use of microorganisms in fermentative processes or through the direct use of enzymes in the catalysis of the toxic element transformation reaction [1, 10].

As it was previously mentioned, lipases modifies oils through hydrolysis reaction, among others; the triglyceride ester bond breaks in the presence of water during the hydrolysis reaction process and produces glycerol and fatty acids [11]. In addition, the change may happen through transesterification, wherein the alcohol is displaced from the ester by another alcohol; first, the triglyceride converts into diglyceride. Then, the diglyceride converts into monoglyceride, which finally converts into glycerol, thus resulting in a methyl or ethyl ester of each glyceride in each transformation stage [12].

Thus, the aim of the current study was to analyze the effect of cooking oil waste bioremediation through hydrolysis and transesterification (alcoholyze) reactions catalyzed by lipases obtained from orange and commercial wastes. The changes in the composition and cytotoxicity of the oil and of its residue, before and after enzymatic treatment, were herein analyzed.

The soybean oil wastes treated with core and frit lipases through transesterification reaction were less toxic than the untreated oils, thus confirming that cooking oil wastes can be bioremediated using orange lipases.

## Materials and methods

### Lipase obtaining, concentration, determination and identification

The plant lipases used in the current study were obtained from the waste resulting from oranges (‘Pera’ variety cultivated in São Paulo State) processed for concentrated juice production. Three waste fractions were used, namely: frit (peel fragment), peel and core. The samples were processed by mechanical milling, freezing and lyophilization [13–14].

The crude extracts were concentrated according to different methods. After the initial processing, enzyme extracts were concentrated through precipitation using ammonium sulfate (60% saturation), followed by centrifugation, dialysis and lyophilization; as well as using acetone (acetone:sample, at the ratio 1:4, v/v, respectively), followed by centrifugation and evaporation. They were also subjected to microfiltration through centrifugation using the 10 KDa Centrifugal filter (Millipore^®^) membrane.

The orange lipase activities were measured before and after each stage according to the titration methods used emulsified olive oil as substrate to measure hydrolysis activity [15]; and used oleic acid and ethanol as substrate (1 mol acid: 5 mol alcohol) to measure esterification activity [16]. Total proteins were quantified according to the Biuret method [17].

The lipase activity was identified through zymography by using MUF-butyrate substrate (Sigma Aldrich^®^) [18]. The samples were prepared by resuspension in Tris buffer (0.05 M, pH 7.4) containing CHAPS (4%) and protease inhibitor (Sigma-Aldrich 1mM); then, they were homogenized in probe and centrifuged [19]. Different polyacrylamide concentrations, ranging from 5 to 15%, were tested in order to prepare the 10% native polyacrylamide gel. The gel was prepared with 4 mL distilled water, 2.5 ml HCl Tris buffer (0.5 M, pH 8.8), 3.35 mL acrylamide/bis-acrylamide (30/0.8%), 5 μL TEMED, and 50 μL ammonium persulfate. Each gel well was added with 20 μL of each sample and the run was performed at 150 volts. After the electrophoresis, the gel was placed in a vessel containing 100 mL Tris buffer (50 mM, pH 8.0), 2% triton X-100 and 10 μM methylumbelliferyl (MUF) -butyrate for 15 min, at 37°C. The bands were analyzed under ultraviolet light (UV) to allow finding lipase in the extracts. The results were expressed as mean ± standard deviation (SD) and analyzed through Tukey test; all pairs of groups were compared through One-Way ANOVA (non-parametric); p <0.05 was considered statistically significant, whereas p <0.0001 was considered highly significant. The bands were quantified in the Image J 1.15 software. The statistical analysis was performed in the GraphPad Prism 6 software.

### Preparing the cooking oil waste

The cooking oil waste was collected under controlled conditions in order to assure the reproducibility of the experiment used the conventional soybean oil, which was subjected to heating cycles at 200°C, totaling 35 heating hs [20].

The untreated oil waste was analyzed for further comparison to the enzymatic treatment waste. The unheated oil (crude oil), with and without enzymatic treatment, was also assessed in order to assure that the differences found in the comparison would reflect the enzymatic treatment in the oil waste, thus avoiding false positives due to the normal enzyme-oil reaction.

### Treatment of the crude soybean oil and its waste

The orange-waste lipases were tested in two modification reactions using two substrates, namely: the soybean oil waste and the crude soybean oil (without heating). Commercial porcine pancreatic lipase (Sigma-Aldrich^®^) and *Candida antarctica* lipase (Novozymes^®^) were also subjected to performance analysis for comparative purposes. The treatments were organized as described in Table 1. Each reaction type presented 10 different treatments.

**Table 1:** Bioremediation-study treatments used in titrimetric tests, as well as in compound identification through gas chromatography and cell culture.

Reaction	Treatment	Enzyme extract	Substrate
Hydrolysis	**TH1**	Peel, ‘Pera’ variety, [60%]	Crude oil
	**TH2**	Peel, ‘Pera’ variety, [60%]	Waste
	**TH3**	Frit, ‘Pera’ variety, [60%]	Crude oil
	**TH4**	Frit, ‘Pera’ variety, [60%]	Waste
	**TH5**	Core, ‘Pera’ variety, [60%]	Crude oil
	**TH6**	Core, ‘Pera’ variety, [60%]	Waste
	**TH7**	Sigma Lipase	Crude oil
	**TH8**	Sigma Lipase	Waste
	**TH9**	Novozyme Lipase	Crude oil
	**TH10**	Novozyme Lipase	Waste
Transesterification	**TT1**	Peel, ‘Pera’ variety, [60%]	Crude oil
	**TT2**	Peel, ‘Pera’ variety, [60%]	Waste
	**TT3**	Frit, ‘Pera’ variety,[60%]	Crude oil
	**TT4**	Frit, ‘Pera’ variety,[60%]	Waste
	**TT5**	Core, ‘Pera’ variety,[60%]	Crude oil
	**TT6**	Core, ‘Pera’ variety, [60%]	Waste
	**TT7**	Sigma Lipase	Crude oil
	**TT8**	Sigma Lipase	Waste
	**TT9**	Novozyme Lipase	Crude oil
	**TT10**	Novozyme Lipase	Waste

#### Hydrolysis

The (‘Pera’ variety) orange waste extracts and the commercial porcine pancreatic lipase (Sigma-Aldrich^®^) were used in the hydrolysis of crude soybean oils and soybean oil wastes using 5% lipase extract over the reaction medium volume. Crude oil- and waste-reagent blanks were prepared [21]. The hydrolysis rate was calculated by taking into consideration the NaOH volume used in the titration (mL), multiplied by the concentration and molar mass of the base. The resulting value was then divided by the oil mass (g) and multiplied by the oil saponification index; then, the result was multiplied by 100 in order to be expressed as percentage.

The analyses were performed in duplicate, whereas the hydrolysis data analyzed through acid titration were compared through analysis of variance (ANOVA) and subjected to Tukey test, at 5% probability level, in the Assistat 7.7 beta software. The formed FAE (fatty acid esters) were analyzed through gas chromatography as described below.

#### Transesterification

The (‘Pera’ variety) orange waste extracts and the commercial porcine pancreatic lipase (Sigma-Aldrich^®^) were used in the alcoholysis of the crude soybean oil and soybean oil waste, using 5% lipase extract over the reaction medium volume. The system was incubated for 72 hs [22]. Crude oil- and waste-reagent blanks were prepared. The analyses were performed in duplicate; the identification and quantification of FAE (fatty acid ethyl esters) were performed as described below.

### The identification and quantification of FAE

The samples were analyzed by gas chromatography with flame ionization detector (GC-FID) and by gas chromatography with mass spectrometry (GC/MS) in order to check whether the FAE profile changed in the different treatments. These techniques allowed checking whether there was difference between the crude and heated (waste) oils; whether the bioremediation processes actually modified the oils and, finally; what reaction and enzyme extract most changed the oil wastes.

Samples from the hydrolysis reaction were prepared in a 25mL falcon tube added with 100 μL of sample and 1.5 mL of 0.5 M NaOH (diluted in methanol); the mixture was subjected to heating at 100°C for 5 min. The tube was cooled and 4 mL of 14% BF3-MeOH esterifier—Boron trifluoride in methanol (Sigma-Aldrich^®^) was added to it; the mixture was again subjected to heating at 100°C for 5 min and, then, it was cooled. The resulting esters (fatty acid methyl ester) were extracted by adding 2 mL heptane and 5 mL saturated sodium chloride solution to the mixture, which was homogenized in vortex for 2 min; after the phases were separated, the supernatant was analyzed [23].

The FAE (fatty acid methyl esters and fatty acid ethyl esters) profile in the samples was characterized using Shimadzu GC-FID (GC-2010) and Shimadzu GC-MS (QP-50), operating with electron impact (70 eV), both equipped with OV-5 fused silica capillary column (Ohio Valley Specialty Chemical, Inc.; 30.0m x 0.25mm x 0.25μm). The temperature program started at 180°C for 10 min, with heating ramp 5°C.min^-1^ up to 280°C, remaining for 10 min. The injector temperature was 230ºC, the detector temperature was 280ºC; helium was used as carrier gas at the flow 1 mL.min^-1^; the sample injection volume was 1μL; split 1/20.

The FAE were quantified through GC-FID, according to the area normalization method. The identification of fatty acid esters was accomplished by comparing the retention time between samples and the fatty acid ethyl esters and fatty acid methyl esters standards (Sigma-Aldrich^®^), as well as by comparing the mass spectrum using the NIST (Nist62.lib and Wiley139.lib) and WebBook databases (http://webbook.nist.gov/chemistry/).

The results of the analysis of the fatty acids profile in samples were compared through multivariate principal component analysis (PCA); the hierarchical cluster analysis (Cluster) was performed in the XLSTAT software—version 2017.3 (Addinsoft, France).

### Cytotoxicity

The cytotoxicity assay was conducted according to the method described in the ISO 10993–12: 2016. Fibroblasts (NIH-3T3) were routinely kept under classical cell culture and humidity conditions, at 37°C.The cells were previously seeded into 96 well-plates at 5.000 cells/well. Next, they were treated at semi-confluence using 3 different dosages, with 5 repetitions each. After 24 hs, the cells were subjected to MTT approach for additional 3 hs. Thereafter, the MTT solution was removed and 100µl of DMSO was added to it in order to solubilize the dye formed by viable cells. Then, the absorbance was measured at 570 nm using a microplate reader (SYNERGY-HTX multi-mode reader, Biotek, USA). Importantly, the hydrophobic compounds from the oil waste were solubilized in 1% arabic gum and it was used in all cytotoxicity approaches to deliver the compounds, since the cell culture medium present high hydrophilicity.

#### Reaction medium standardization

The samples from the bioremediation experiments were made up of lipids; as the cell culture medium was aqueous, the contact between lipids was just superficial, fact that could compromise the reliability of the results found in the cell culture assays. Therefore, different substances were tested in order to reduce the surface tension in the culture medium, namely: Tween 20, Triton-X (1% over the culture medium volume) and gum arabic (3% over the culture medium volume), by applying the same parameters used in the cytotoxicity assay described above. The substance promoting the greatest contact in the reaction media (oil samples) and lower cytotoxicity was selected.

### Planning the experimental blocks

The experiments were conducted on 96-well (8x12) tissue culture plates, using 60 core wells with borders. Each plate was divided in 10 columns containing 6 wells; each treatment was placed in a column, with 6 repetitions. The total reaction medium volume in each well was set at 250 μL.

A single treatment consisting of enzyme extract x reaction was tested on each plate, i.e., 8 plates were used, namely: Plate 1—Frit Lipase / Transesterification; Plate 2—Peel Lipase / Transesterification; Plate 3—Core Lipase / Transesterification; Plate 4—Commercial Sigma Lipase / Transesterification; Plate 5—Frit lipase / Hydrolysis; Plate 6—Peel Lipase / Hydrolysis; Plate 7—Core Lipase / Hydrolysis; Plate 8—Commercial Sigma Lipase / Hydrolysis.

The two substrates, the crude oil and the oil waste were tested on each plate, at 3 different concentrations each. Three (3) controls were also included on each plate; therefore, each plate comprised 9 treatments, namely:

- Control 1 (CTRL): Reagent blank consisting of 100 μL tissue culture medium with serum and 150 μL serum-free reaction medium, in order to assure serum viability;
- Control 2 (BTC): Crude oil consisting of 100 μL tissue culture medium with serum, 25 μL gum arabic (10%), 10 μL crude oil and 110 μL serum-free reaction medium, for comparison between crude oil with and without enzymatic treatment, and between crude oil and oil waste;
- Control 3 (BTR): Oil waste consisting of 100 μl tissue culture medium with serum, 25 μl gum arabic (10%), 10 μL oil waste and 110 μL serum-free reaction medium, for comparison between the oil waste with and without enzymatic treatment, and between crude oil and oil waste;
- Enzyme extract X, X reaction in crude oil—volume 1 (TC1) consisting of 100 μL tissue culture medium with serum, 25 μL gum arabic (10%), 5 μL enzyme extract X, X reaction in crude oil and 120 μL serum-free reaction medium, for comparison between different concentrations, different substrates and non-enzymatically treated crude oil;
- Enzyme extract X, X reaction in crude oil—volume 2 (TC2) consisting of 100 μL tissue culture medium with serum, 25 μL gum arabic (10%), 10 μL enzyme extract X, X reaction (crude oil) and 115μL serum-free reaction medium, for comparison between different concentrations, different substrates, and non-enzymatically treated crude oil;
- Enzyme extract X, X reaction in crude oil—volume 3 (TC3) consisting of 100 μL tissue culture medium with serum, 25 μL gum arabic (10%), 15 μL enzyme extract X, X reaction (crude oil) and 120 μL serum-free reaction medium, for comparison between different concentrations, different substrates, and non-enzymatically treated crude oil;
- Enzyme extract X, X reaction in oil waste—volume 1 (TR1) consisting of 100 μL tissue culture medium with serum, 25 μL gum arabic (10%), 5 μL enzyme extract X, X reaction (oil waste) and 120 μL serum-free reaction medium, for comparison between different concentrations, different substrates and non-enzymatically treated oil waste;
- Enzyme extract X, X reaction in oil waste—volume 2 (TR2) consisting of 100 μL tissue culture medium with serum, 25 μL gum arabic (10%), 10 μL enzyme extract X, X reaction (oil waste) and 115 μL serum-free reaction medium, for comparison between different concentrations, different substrates and non-enzymatically treated oil waste;
- Enzyme extract X, X reaction in oil waste—volume 3 (TR3) consisting of 100 μL tissue culture medium with serum, 25 μL gum arabic (10%), 15 μL enzyme extract X, X reaction (oil waste) and 120 μL serum-free reaction medium, for comparison between different concentrations, different substrates and non-enzymatically treated oil waste.

## Results

### Lipase obtaining, concentration, determination and identification

Different methods applied to the concentration of plant extract-derived lipases showed that the concentration through precipitation using ammonium sulfate, followed by dialysis and lyophilization, showed the highest lipase activity in U/g; the frit extract reached 50.86 U/g. The acetone and microfiltration concentrations have reduced the lipase activity in the extracts; the peel microfiltration reached 0 U/g. (Table 2).

**Table 2:** Concentration of lipase extracts obtained from orange wastes.

Sample	Method	U of lipase/g of extract	TP** mg/g	U/mg of TP*
**Frit**	Crude extract	06.311±017	5.87	1.075±0.02
	Precipitation/lyophilization	50.862±1.45	54.31	0.936±0.02
	80% Acetone	04.979±0.29	38.36	0.129±0.01
	Microfiltration	16.916±0.57	48.67	0.347±0.01
**Peel**	Crude extract	05.577±0.35	5.93	0.940±0.05
	Precipitation/lyophilization	19.426±0.30	27.48	0.706±0.01
	80% Acetone	03.527±0.35	31.57	0.111±0.01
	Microfiltration	0	36.46	0
**Core**	Crude extract	04.232±0.35	3.76	1.125±0.05
	Precipitation/lyophilization	29.298±0.59	55.75	0.525±0.01
	80% Acetone	02.198±0.15	28.95	0.075±0.01
	Microfiltration	0		0

* The results were expressed as mean ± standard deviation

** TP = total proteins

**Fig 1:**
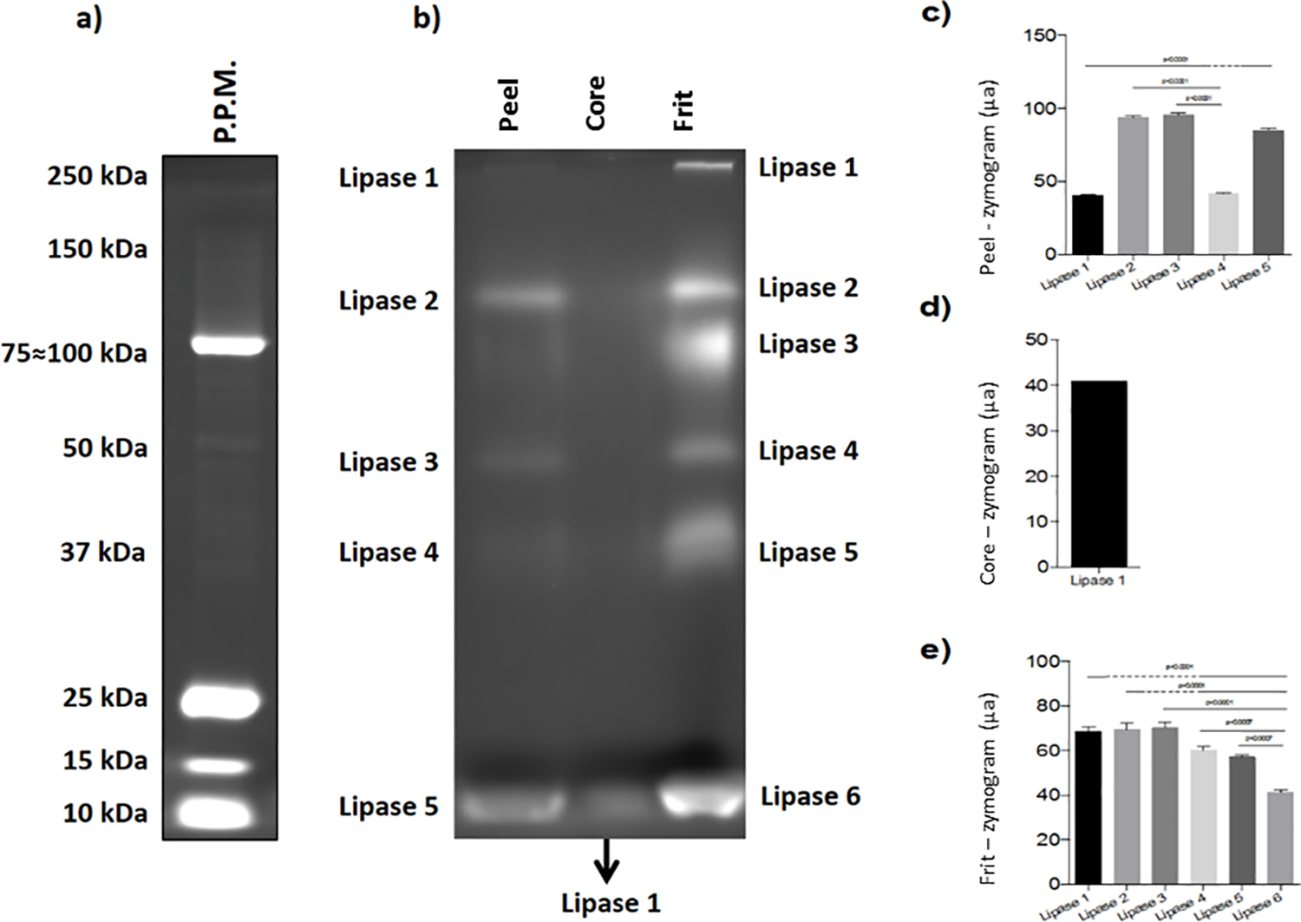
Zymogram of lipases obtained from orange wastes; a) zymogram of the standard; b) zymogram of orange peel, core and frit lipases; c), d) and e) quantification of zymography bands of peel, core and frit lipases, respectively.

Therefore, the selected lipase extract concentration method used in the current study was the precipitation method through ammonium sulfate, followed by dialysis and lyophilization. For all the other experiments the extracts concentrated with ammonium sulfate at 60% saturation were used. The obtained lipases were analyzed into varying concentrations of polyacrylamide gels in order to generate the lipase zymogram. Thereafter, our results showed that the concentration 10% was the most effective in separating lipases and the most effective for proteins separation, based on their molecular weight. Based on the zymogram, Fig 1 shows that the peel fraction presented 5 different bands whose weights ranged from 10 to 250 kDa, whereas the core fraction presented a single 10 kDa band; and the frit fraction presented 6 bands whose weight ranged from 10 to 250 kDa.

### Treatment applied to the crude soybean oil and to its waste

**Fig 2:**
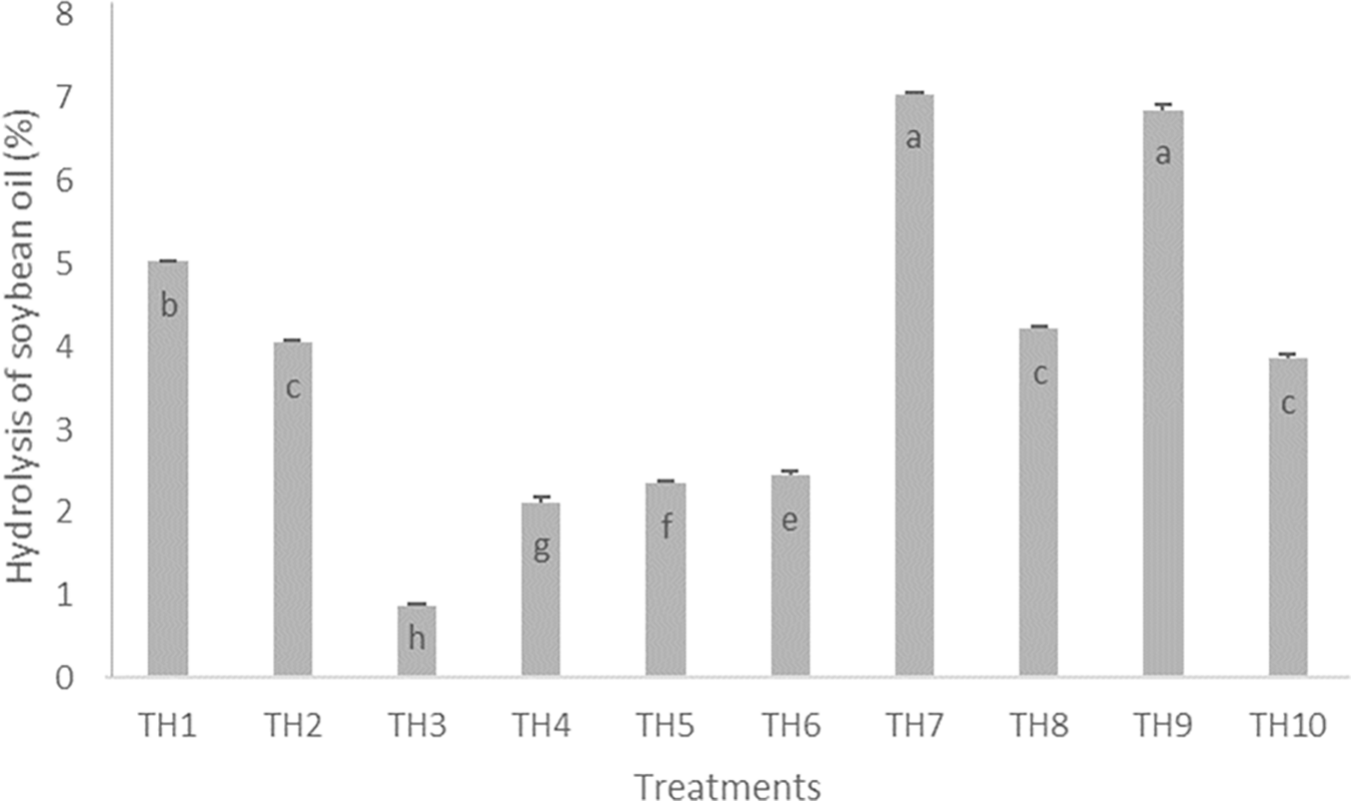
Hydrolysis of soybean oil catalyzed by commercial and homemade lipases. Wherein: 1 = TH1 / core / crude soybean; 2 = TH2 / core / soybean waste; 3 = TH3 / peel / crude soybean; 4 = TH4 / peel / soybean waste; 5 = TH5 / frit / crude soybean; 6 = TH6 / frit / soybean waste; 7 = TH7 / Lipozyme / crude soybean; 8 = TH8 / Lipozyme / soybean waste; 9 = TH9 / Sigma / crude soybean; and, 10 = TH10 / Sigma / soybean waste. The values followed by same letter do not differ statistically by Skott Knott test (p≤0,05).

Commercial Sigma and orange waste lipases showed higher release of FAE than the reference samples, namely: crude soybean oil blank and soybean oil waste blank (Fig 2). In addition, it was possible seeing that the experiments using crude soybean oil and soybean oil waste showed different FAE releases in the core and commercial lipase treatments. These results indicate that the lipolytic extracts were able to modify the soybean oil, both when it was raw and after it was subjected to prolonged heating. Thus, it was assumed that orange lipases have potential to modify vegetable oils for bioremediation purposes, as well as that the compounds deriving from the oil heating process did not influence the catalysis power of the plant lipases.

#### Identifying and quantifying of chemical composition

The fatty acid ester (FAE) profile analyses were conducted through gas chromatography and showed that all lipase extracts were able to catalyze the transesterification reaction of crude soybean oil and of its residue when ethanol was used as solvent, since FAE (fatty acid ethyl esters) formed in all samples treated with lipases with the main FAE identified for orange lipases was ethyl oleate (relative% ranging from 88.11 to 94.90%), while for the lipozyme and sigma lipases were ethyl oleate (27.56 to 48.25%), ethyl elaidate (30.49 to 38.90%) and ethyl palmitate (9.70 to 11.37%) (S1 Fig).

**Fig 3:**
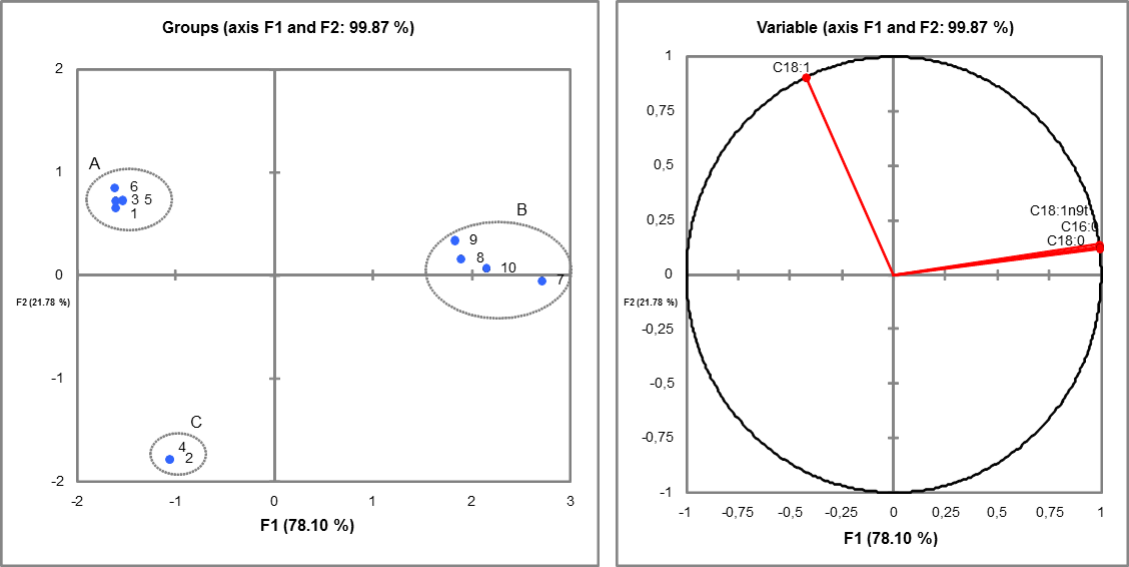
PCA applied to the chemical composition (fatty acids) of the transesterification reaction samples. Wherein: 1 = TT1 / core / crude soybean; 2 = TT2 / core / soybean waste; 3 = TT3 / peel / crude soybean; 4 = TT4 / peel / soybean waste; 5 = TT5 / frit / crude soybean; 6 = TT6 / frit / soybean waste; 7 = TT7 / Lipozyme / crude soybean; 8 = TT8 / Lipozyme / soybean waste; 9 = TT9 / Sigma / crude soybean; and, 10 = TT10 / Sigma / soybean waste.

The PCA applied to the chemical composition of the fatty acid expressed 99.87% of the variance in the first two principal components; the PCA1 (F1) accounted for 78.10% of the total variance, whereas the PCA2 (F2) accounted for 21.78% of it. The variables provided by the PCA allowed identifying the separation of the samples into three groups set according to oleic acid (C18:1n9c) (group A), palmitic acid (C16:0), stearic acid (C18:0) and elaidic acid (C18:1n9t) (group B); and one group (C) that showed no correlation with any of the fatty acids identified in the experiment (Fig 3).

**Fig 4:**
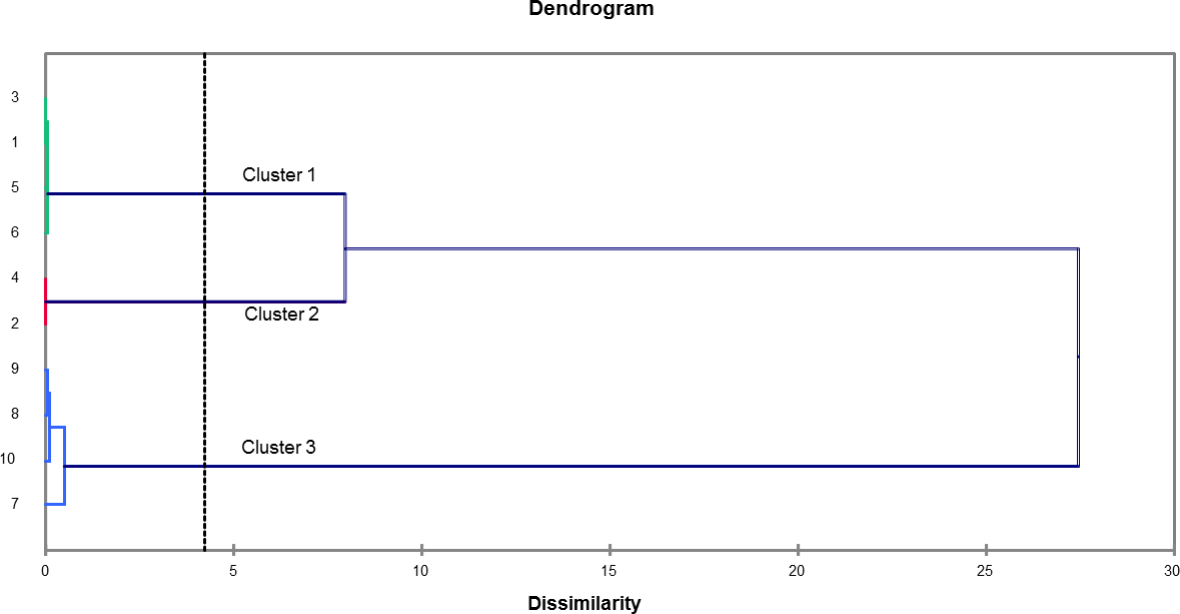
Dissimilarity dendrogram built through the hierarchical clustering analysis (cluster analysis) of lipase-catalyzed transesterification samples. Wherein: 1 = TT1 / core / crude soybean; 2 = TT2 / core / soybean waste; 3 = TT3 / peel / crude soybean; 4 = TT4 / peel / soybean waste; 5 = TT5 / frit / crude soybean; 6 = TT6 / frit / soybean waste; 7 = TT7 / Lipozyme / crude soybean; 8 = TT8 / Lipozyme / soybean waste; 9 = TT9 / Sigma / crude soybean; and, 10 = TT10 / Sigma / soybean waste.

**Fig 5:**
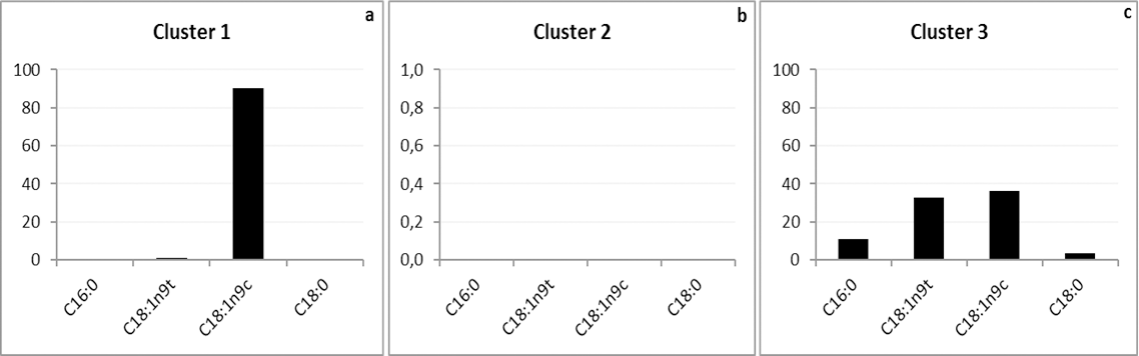
Significance of the most important FAE for Clusters 1 to 3 of the lipase-catalyzed transesterification samples (Axis: X- fatty acids; Y—% of the total variance; analyzes performed with the mean of the concentrations measured between the replicates).

Similar results were found in hierarchical cluster analysis (Cluster). The dendrogram (Fig 4) build through the Cluster analysis of the dissimilarity matrix (Pearson’s correlation coefficient) showed three main clusters (Clusters 1, 2 and 3). According to Fig 4, Cluster 1, which was represented by samples 1, 3, 5 and 6 (TT1 / Core / crude soybean; TT3 / peel / crude soybean; TT5 / frit / crude soybean; and TT6 / frit / soybean waste, respectively), showed the highest relative oleic acid ratio (C18:1n9c) (Fig 5A) when it was compared to the other samples. Cluster 2, which was represented by samples 2 (TT2 / core / soybean waste) and 4 (TT4 / peel / soybean waste), did not show fatty acids in its chemical composition (Fig 5B). Cluster 3, which was represented by samples 7, 8, 9 and 10 (TT7 / Lipozyme / crude soybean; TT8 / Lipozyme / soybean waste; TT9 / Sigma / crude soybean; and TT10 / Sigma / soybean waste, respectively) stood out because the samples showed higher production of palmitic acid (C16:0), stearic acid (C18:0) and elaidic acid (C18:1n9t) FAE (Fig 5C) when they were compared to the other samples.

The hydrolysis reaction results showed that all lipase extracts were able to catalyze the hydrolysis reaction of the crude soybean oil and of its residue, since there was fatty acids release in all the enzyme-treated samples. The main FAE (fatty acid methyl esters) identified were methyl palmitate, methyl linoleate, methyl oleate and methyl stearate.

**Fig 6:**
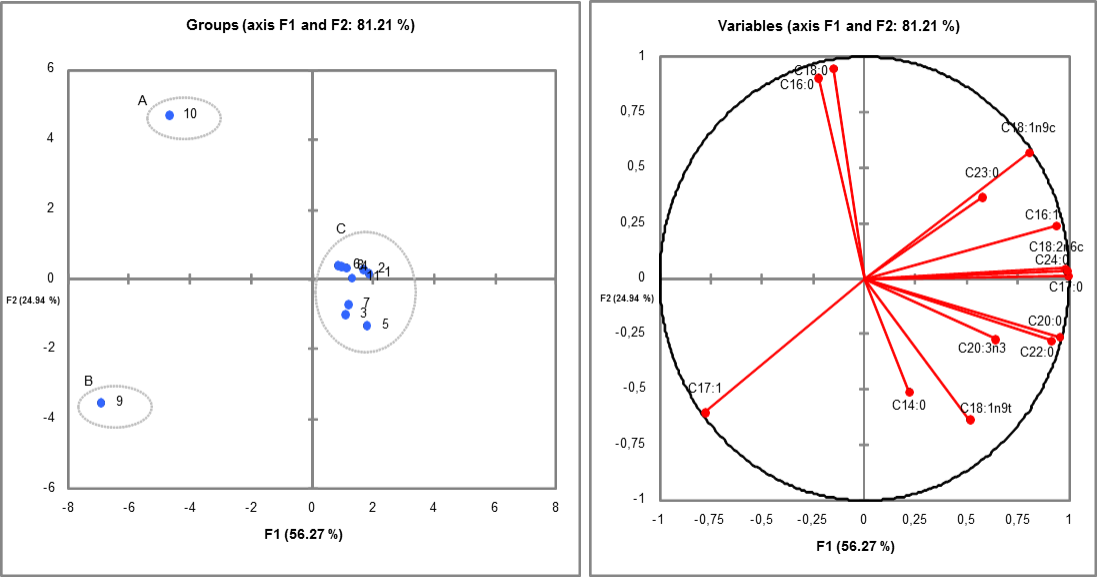
PCA applied to the chemical composition (fatty acids) of the lipase-catalyzed hydrolysis reaction samples. Wherein: 1 = TH1 / core / crude soybean; 2 = TH2 / core / soybean waste; 3 = TH3 / peel / crude soybean; 4 = TH4 / peel / soybean waste; 5 = TH5 / frit / crude soybean; 6 = TH6 / frit / soybean waste; 7 = TH7 / Lipozyme / crude soybean; 8 = TH8 / Lipozyme / soybean waste; 9 = TH9 / Sigma / crude soybean; and, 10 = TH10 / Sigma / soybean waste.

The Principal Component Analysis (PCA) applied to the chemical composition of the fatty acids expressed 81.21% variance in the first two principal components; the PCA1 (F1) accounted for 56.27% of the total variance, whereas the PCA2 (F2) accounted for 24.94% of it. The variables provided by the PCA allowed identifying the separation of the samples into three distinct groups: group A (sample 10) was set according to palmitic acid (C16:0) and stearic acid (C18:0); group B (sample 9) was set according to the cis-10-heptadecanoic acid (C17:1); and group C (samples: 1,2,3,4,5,6,7,8) was set according to the other fatty acids (Fig 6).

**Fig 7:**
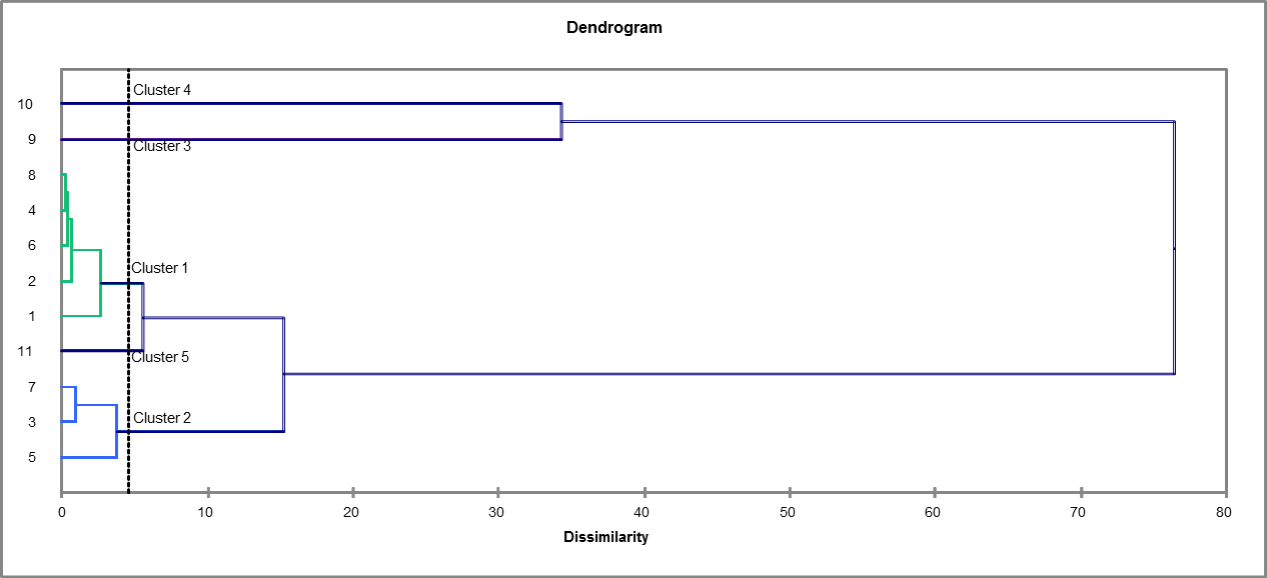
Dissimilarity dendrogram built through the hierarchical cluster analysis of the lipase-catalyzed hydrolysis reaction samples. Wherein: 1 = TH1 / core / crude soybean; 2 = TH2 / core / soybean waste; 3 = TH3 / peel / crude soybean; 4 = TH4 / peel / soybean waste; 5 = TH5 / frit / crude soybean; 6 = TH6 / frit / soybean waste; 7 = TH7 / Lipozyme / crude soybean; 8 = TH8 / Lipozyme / soybean waste; 9 = TH9 / Sigma / crude soybean; and, 10 = TH10 / Sigma / soybean waste.

**Fig 8:**
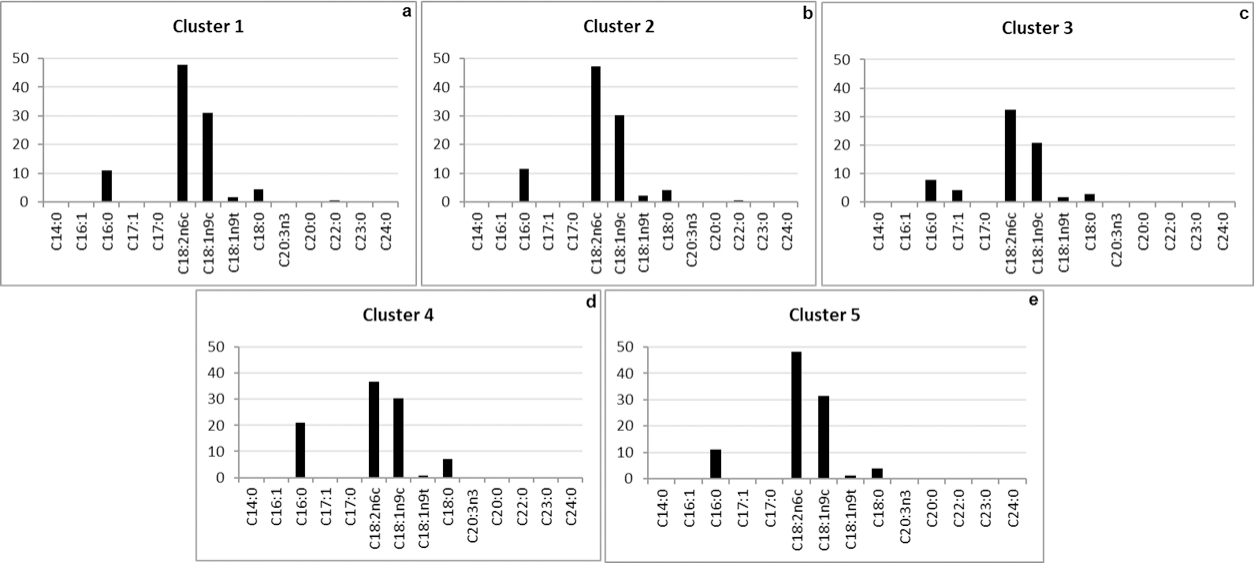
Significance of the most important FAE for Clusters 1 to 5 of the of lipase-catalyzed hydrolysis samples. (Axis: X- fatty acids Y—% of the total variance; analyzes performed with the mean of the concentrations measured between the replicates).

The hierarchical cluster analysis produced five main clusters (Fig 7); the third and fourth clusters (Clusters 3 and 4) presented greater dissimilarity (difference) than the others (Clusters 1, 2 and 5). This difference happened because Cluster 3, which was represented by sample 9 (TH9 / Lipozyme / soybean waste), showed the highest relative (4.23%) cis-10-heptadecanoic acid ratio (C17:1) (Fig 8C) than the other samples (relative ratio ranging from 0 to 0.09%). Cluster 4, which was represented by sample 10 (Treatment 10 / Sigma / Crude soybean), showed the highest (20.93%) palmitic acid ratio (C16:0), whereas the other samples presented relative ratio ranging from 7.69% to 11.63%. The other clusters (1, 2 and 5), which were represented by the other samples (1 to 8 and 11), showed similar chemical composition (Figs 7 and 8A, 8B and 8E).

### Cytotoxicity

**Fig 9:**
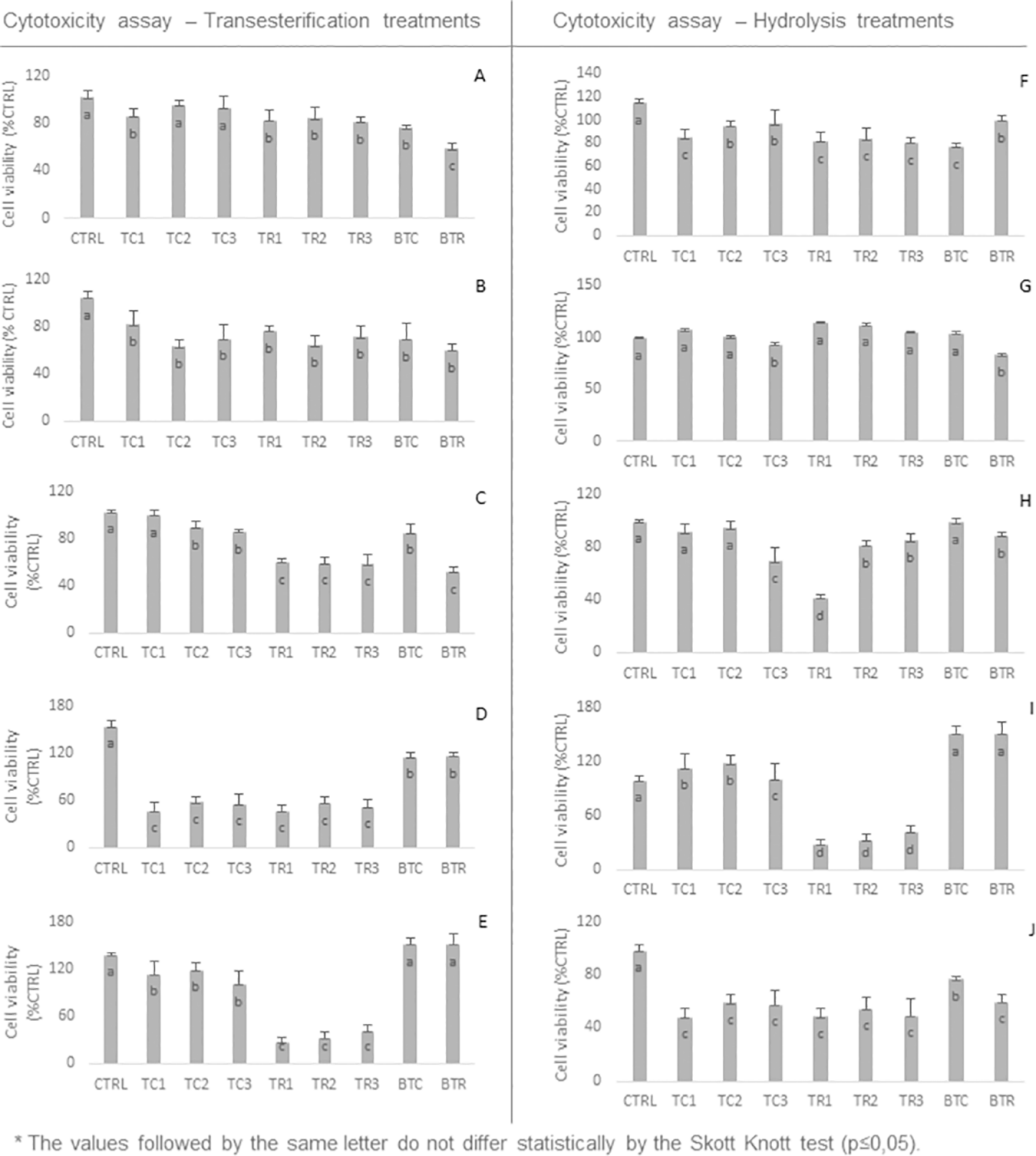
Cytotoxicity assay conducted through transesterification and hydrolysis. Wherein: Lipases: A–core (TT1 and TT2); B–peel (TT3 and TT4); C–frit (TT5 and TT6); D–Novozymes (TT7 and TT8); E–Sigma (TT9 and TT10) through hydrolysis; and Lipases: F–core (TH1 and TH2); G–peel (TH3 and TH4); H–frit (TH5 and TH6); I–Sigma (TH7 and TH8); J–Novozymes (TH9 and TH10); CTRL—culture medium control; TC1-3: enzyme-treated crude oil, 5 to 15μL; TR1-3: enzyme-treated oil waste, 5 to 15 μL; BTC—soybean oil control; BTR—soybean oil waste control). The values followed by same letter do not differ statistically by Skott Knott test (p≤0,05).

The lipid wastes dispersed in the environment primarily come into contact with the living beings through the epidermis. Accordingly, cytotoxicity tests were conducted *in vitro* using fibroblasts in order to mimic the effect of the samples on living cells. In addition, fibroblasts are suggested and the most used to analyze the molecules toxicity [23]. Over the last decades, fibroblast assays have been useful and reliable in the analysis of skin irritation caused by numerous agents [24–25], analyzed the role played by surfactants in skin irritation and found that assays conducted *in vitro* were able to replicate the results *in vivo*. Thus, Fig 9 shows the results of the cytotoxicity analysis conducted in the soybean oil and in the soybean oil waste modified through transesterification and hydrolysis reactions. The reagent blank control (CTRL) showed the highest MTT activity, thus indicating higher viable cell rate. In addition, it can be used to validate our assays since these cells were kept under classical culture conditions by respecting the nutrients and bovine fetal serum amounts. Subsequently, we showed that the cell viability rate in response to the soybean oil control (BTC) was higher than that of the soybean oil waste (BTR); thus evidencing that the soybean oil waste is even more cytotoxic than the soybean oil.

Lately, we have also identified the cytotoxic effect of transesterification sub-products, namely: the soybean oil treatments using core lipase (TC1, TC2 and TC3) showed higher cell viability than the BTC, whereas the soybean oil waste treatments using core lipase (TR1, TR2 and TR3) showed higher cell viability than the BTR (Fig 9A). The soybean oil and soybean oil waste treatments using peel lipase did not show significance in cell viability when they were compared to the blank treatments (Fig 9B). The soybean oil treatments using frit lipase showed higher cell viability than the BTC, whereas the soybean oil waste treatments using frit lipase showed higher cell viability than the BTR; however, the differences were not significant as reported (Fig 9C). The soybean oil and soybean oil waste treatments using commercial lipases (Sigma and Novozymes) showed lower cell viability than the BTC and BTR (Fig 9D and 9E). It indicates that the transesterification changes catalyzed by core and frit lipases decreased the cytotoxicity of both the soybean oil and its waste, whereas the transesterification change catalyzed by commercial lipases increased the cytotoxicity of both the soybean oil and its waste.

According to the results of the hydrolysis cytotoxicity assay, the soybean oil treatments using core lipase (TC2 and TC3) showed higher cell viability than the BTC, whereas the soybean oil waste treatments using core lipase (TR1, TR2 and TR3) showed higher cell viability than the BTR (Fig 9F). The soybean oil and soybean oil waste treatments using peel lipase did not show significance in cell viability when they were compared to the blank treatments (Fig 9G). The soybean oil treatment using frit lipase (TC1) showed higher cell viability than the BTC, whereas the soybean oil waste treatments using frit lipase did not show differences in cell viability when they were compared to the BTR (Fig 9H). The soybean oil and soybean oil waste treatments using commercial lipases (Sigma and Novozymes Companies) showed lower cell viability than the BTC and BTR (Fig 9I and 9J). It indicates that the core lipase-catalyzed hydrolysis change decreased the cytotoxicity of both the soybean oil and its residue, whereas the commercial lipase-catalyzed hydrolysis change increased the cytotoxicity of both the soybean oil and its residue.

Briefly, it suggests trans-esterification changes catalyzed through core and frit lipases decreased the cytotoxicity of the soybean oil waste. However, the transesterification and hydrolysis changes catalyzed through commercial lipases were not effective in the bioremediation of soybean oil wastes, since the viable cell rate in the treated waste was equal to or lower than that of the untreated waste. In addition, the results of the transesterification treatments were different from those of the hydrolysis treatments.

## Discussion

The low lipase activity resulting from acetone concentration corroborates other studies about plant lipases [26], overall, plant lipases did not tolerate high organic solvent concentrations. However, the low lipase activities resulting from the microfiltration concentration can be explained through the membrane porosity, which may not have been suitable for the lipases, since these enzymes are new and there was no information about their size; thus, the porosity was selected according to the recommendation for various enzymes. Comparatively, frit and peel fraction extracts presented similar profiles, whereas the core extract showed a distinct one. It can be anatomically explained, since the frit and the peel correspond to the fruit epicarp, whereas the core corresponds to the mesocarp [27]. Our results bring valuable information about the anatomic-based distribution of lipases in orange. Thus, we have reported the presence of lipase isoforms in these extracts in biochemical characterization [13]. It is known that orange-derived lipases differ from other vegetable sources in weight and isoform variety, since the plant lipases in most studies weight 36–40 kDa (lipases obtained from rice, Barbados nut and *Arabidopsis* ssp.) up to 60 kDa (lipids obtained from castor bean and turnip) [26, 28–30]. It is worth taking into consideration that the lipases detected in the zymogram showed higher affinity for short-chain fatty acids, since MUF-butyrate was used. Thus, there may be other lipases with affinity for other fatty acids, such as the medium- and long-chain ones. Thus, the presence of lipases with affinity for different substrates could be investigated in zymograms performed according to methodologies using substrates of different chain sizes in hydrolysis and synthesis reactions [31].

In addition, the zymogram preparation comprised samples concentrated through precipitation using 60% ammonium sulfate, followed by centrifugation and lyophilization. It was evidenced that the lipases found in these extracts kept the catalytic activity after these processes, thus confirming that these protein concentration methods are suitable for the concentration of these enzymes.

It was possible concluding that both the commercial and the home-made lipases obtained from the herein tested orange wastes can be used to modify the crude soybean oil and its residue through hydrolysis and transesterification reactions.

Therefore, it was concluded that the core and frit lipases were effective in the bioremediation of soybean oil wastes through transesterification (Fig 9A and 9C). The results of the transesterification reaction corroborate those found by other authors [32] who analyzed soybean oil samples modified through transesterification using ethanol and found increased BEAS-2B (human bronchial epithelial cells) proliferation. However, we suggest evaluating these effects on other cell lines since the skin tissue presents more types of cellular phenotypes than fibroblasts. Another point to be explored lies on the variability among individuals. However, approaches *in vivo* are necessary in order to address this issue.

In addition, the results of the cytotoxicity analysis were compared to the results of the analysis conducted in the chemical profile of the samples treated with orange waste lipases. It was possible seeing that the treatments showing decreased cytotoxicity, as well as transesterification catalyzed by core and frit lipases (Fig 9A and 9C), were ranked in the same statistical group according to the principal component analysis of the fatty acids profile. Group A showed the highest oleic acid concentration, whereas group B showed the highest palmitic acid, stearic acid and elaidic acid concentrations (Fig 3). In addition, as it happened in the cytotoxicity assays, the FAE profile found in the transesterification treatments differed from that found in the hydrolysis treatments. It confirms that the transesterification and hydrolysis based modifications resulted in different products.

The food industry has increasingly sought waste disposal solutions able to be less environmentally damaging. Bioremediation stands out among these solutions, since it uses biological elements to neutralize or reduce toxicity [9]. Thus, high lipid-content wastes bioremediated through microbial lipases have been reported in studies who investigated the bioremediation of cooking oil wastes using lipases produced by *Penicillium chrysogenum* [2], as well the bioremediation of olive oil extraction wastes using lipases produced by *Aspergillus ibericus* and *Aspergillus uvarum* [33]. However, the current study is the first to describe the bioremediation process using lipases from vegetable wastes and it introduces an innovative process, since it presents the possibility of using a waste to solve the problem generated by another waste, therefore contributing to the sustainability of the food production chain.

## Conclusion

The commercial and orange waste lipases tested in the current study can be used to modify crude soybean oil and its waste by means of hydrolysis and transesterification reactions and it seems significantly modulate cell viability. Soybean oil wastes treated with core and frit lipases through transesterification reaction were less toxic than the untreated oils, thus confirming that such wastes can be bioremediated using orange lipases.

## Supporting information

S1 Fig GC- FID chromatogram image of crude (A) and heated–waste (B) oils.
(TIF)
