# Supplementary figures and images for: Bioremediation of cooking oil waste using lipases from wastes
Source: PLoS One. 2017 Oct 26;12(10):e0186246. doi: 10.1371/journal.pone.0186246 (PMC5657992; doi:10.1371/journal.pone.0186246)

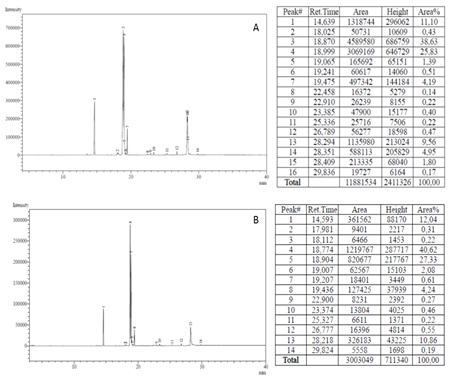

Supplement: S1 Fig — GC- FID chromatogram image of crude (A) and heated–waste (B) oils. (TIF) [file pone.0186246.s001.tif]
